# Minimally Invasive Trans-sacral Canal Plasty for Lumbar Canal Stenosis

**DOI:** 10.7759/cureus.59509

**Published:** 2024-05-02

**Authors:** Seiya Watanabe, Kazuo Nakanishi, Ryo Sato, Yoshihisa Sugimoto, Shigeru Mitani

**Affiliations:** 1 Orthopedics, Kawasaki Medical School, Okayama, JPN

**Keywords:** lumbar canal stenosis, leg pain, low back pain, spinal canal area, trans-sacral canal plasty

## Abstract

Objective

The elderly population is increasing in Japan. Along with the increase in the elderly population, the number of patients with lumbar degenerative diseases is also on the rise. In general, elderly patients tend to have more complications and are at higher risk for surgery. Many elderly people suffer from lumbar degenerative disease. We reviewed our initial experience with trans-sacral canal plasty (TSCP) for patients with lumbar spinal canal stenosis and examined the pertinent literature for this report.

Methods

An analytical observational study was performed on 120 patients with lumbar spinal canal stenosis who underwent TSCP at our single institution from March 2019 to October 2021. These patients had leg pain and/or lower back pain due to degenerative lumbar disease. Patients who had coagulation abnormality, pregnancy, contrast allergy, pyogenic spondylitis, or spinal metastasis were excluded.

Results

Immediately after TSCP, the average Visual Analog Scale (VAS) score for back pain improved from 58.2 to 29.3, and for leg pain from 72.0 to 31.3. Two years after TSCP, the average VAS score for back pain increased slightly and the average score for leg pain remained almost the same. Additional surgery was performed in 37 of 120 (31%) patients who underwent TSCP. The additional surgery group had significantly worse back pain at one and three months postoperatively than the conservative treatment group. The additional surgery group had significantly worse leg pain immediately after TSCP and at one and three months postoperatively than the conservative treatment group. Logistic regression analysis demonstrated that a decreased spinal canal area (OR 0.986, p = 0.039) was associated with additional surgery.

Conclusions

We reviewed the outcomes of TSCP at our hospital. The average VAS score for back pain and leg pain improved. However, 31% of patients who underwent TSCP required additional surgery. It was found that the spinal canal area was a major factor in the need for additional surgery.

## Introduction

The elderly population is increasing in Japan. Along with this increase, the number of patients with lumbar degenerative diseases is also on the rise. In general, elderly patients tend to have more complications and are at higher risk for surgery. Indications for spinal minimally invasive endoscopic procedures such as microendoscopic discectomy and percutaneous endoscopic discectomy have also increased [1-3]. These conventional surgeries approach the cauda equina and nerve roots from outside of the spinal canal.

Intraspinal canal therapy (ISCT) is a percutaneous approach in which the canal is through the sacral hiatus before entry into the spinal canal. Percutaneous epidural neuroplasty (PEN) is a procedure in which the epidural adhesions are released and saline and steroids are injected [4].

In Japan, epiduroscopy has been clinically applied by anesthesiologists [5-7]. An epiduroscopy study group was established by Japanese anesthesiologists in 2000. Between 2004 and 2016, treatment using epiduroscopy was approved as an advanced medical technology (senshiniryo) by the Japanese Ministry of Health, Labor and Welfare. Percutaneous epidural adhesiolysis was approved by insurance in April 2018. Trans-sacral canal plasty (TSCP) was named by the minimally invasive spinal treatment (MIST) Society [8]. TSCP is one of the minimally invasive spinal surgical treatments. TSCP can be performed under local anesthesia and through a small incision (2-3 mm). Therefore, patients with severe complications can be treated. Conventional lumbar spine surgery is generally approached from the outside of the spinal canal. In contrast, TSCP is approached through the sacral hiatus. This allows for a safe approach even after lumbar spine surgery. We reviewed our initial experience with TSCP for patients with lumbar spinal canal stenosis and examined the pertinent literature for this report.

## Materials and methods

The institutional ethics committee approved this study protocol (approval number 5056-04). The patients consented to have their case data collected and used for publication. From March 2019 to October 2021, 120 patients with lumbar spinal canal stenosis (radicular type: 79 cases, cauda equina type: 31 cases, mixed type: 10 cases) underwent TSCP at our single institution. These patients had leg pain and/or lower back pain due to degenerative lumbar disease. Patients who had coagulation abnormality, pregnancy, contrast allergy, pyogenic spondylitis, or spinal metastasis were excluded. The average age was 72 years old (range 43 to 90). There were 56 men and 64 women. The average follow-up period was nine months (range 1 to 30). There were 25 patients with a history of lumbar spine surgery. Lumbar spondylolisthesis (≥ 5mm) was observed in 16 of 120 patients. Lumbar scoliosis (≥ Cobb 15 degrees) was observed in 20 of 120 patients. As for nerve root blocks, we perform them for diagnostic purposes in cases of stenosis of more than two levels.

We used the Mann-Whitney U test to compare the preoperative Visual Analog Scale (VAS) of back pain and leg pain to the postoperative VAS of back pain and leg pain, and the logistic regression analysis to examine factors that would require additional surgery. The study design is a case-control study. In addition, we also evaluated complications.

Technical note

At our institution, TSCP was performed in the angiography room to facilitate 2-directions fluoroscopy and reduce radiation exposure. We performed a computed tomography (CT) prior to TSCP to confirm the location of the sacral hiatus and lower limit of subarachnoid space and the diameter of the sacral spinal canal. 3DCT was useful to determine the shape of the sacral hiatus. We used the Racz catheter (BREVI-XLTM, Epimed International Inc., Dallas, TX, USA) in cases with a narrow spinal canal and steerable catheter (myeloCath®, Biomedica Healthcare Ltd., Tokyo, Japan) with severe epidural adhesions. After local anesthesia, an 18-gauge needle was inserted into the sacral hiatus and the direction of insertion was confirmed fluoroscopically. We inserted the introducer through a 2mm to 3mm skin incision. The inner cylinder was removed, and a catheter was inserted into the epidural space. Under fluoroscopic guidance, a catheter was inserted ventral to the epidural space and carefully advanced to the location of the stenosis. While confirming pain reproducibility, a contrast medium was injected. A catheter was used to detach the adhesion or saline solution was flushed to detach the adhesion. We could advance the catheter along the nerve root to the foramen and perform an adhesiolysis.

There was a risk of dural injury if the catheter was manipulated too roughly near the thinned dura mater. There is a risk of spinal cord injury if lidocaine enters the subarachnoid space secondary to dural injury. A mixture of steroids and lidocaine was injected in order to reduce inflammation.

Low back pain and leg pain of 120 patients were evaluated by VAS. The clinical outcomes of the conservative treatment group and the group that underwent additional surgery were compared. The conservative treatment group was defined as patients with a sustained therapeutic effect of TSCP. Logistic regression analysis was performed to examine factors leading to additional surgery. Additional surgeries were used as the response variable. Age, gender, selective nerve root block, history of lumbar surgery, intermittent claudication, spondylolisthesis, scoliosis, and the cross-sectional spinal canal area were evaluated as explanatory variables. The cut-off value for the various parameters was set at 5 mm (slip), Cobb 15 degrees (scoliosis), and 80 mm^2^ (the cross-sectional spinal canal area). The cross-sectional spinal canal area was measured by MRI using Enterprise-PACS SYNAPSE5 software. As for lumbar spondylolisthesis, it is believed that an anterior slip of 5 mm will cause symptoms of lumbar spinal canal stenosis [9]. Therefore, a cut-off value of 5 mm was set. Adult scoliosis is defined as a spinal deformity with a Cobb angle in the coronal plane of 10 degrees or greater in a skeletally mature patient. Mild disease is defined as less than 20 degrees [10]. Therefore, the cut-off value for scoliosis was set at 15 degrees. The definition of lumbar spinal canal stenosis is defined as a dural canal area of less than 80 mm^2 ^[11]. Therefore, the cut-off value was set at 80 mm^2^.

Statistical analysis

Mann-Whitney U test was used for the purpose of statistical analysis. P < 0.05 was regarded as significant.

## Results

**Figure 1:**
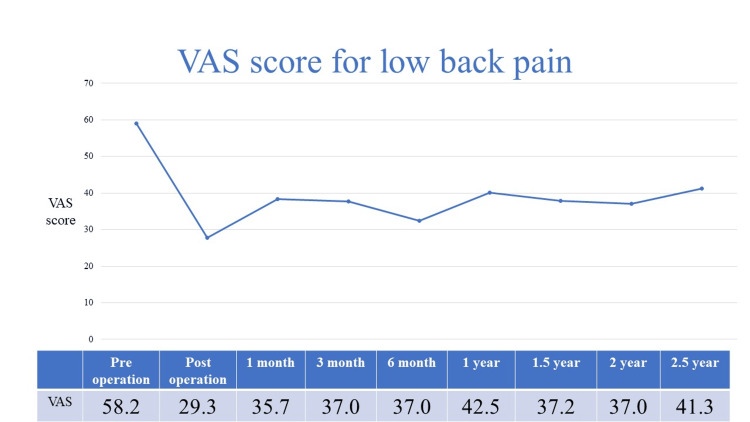
VAS score for low back pain

**Figure 2:**
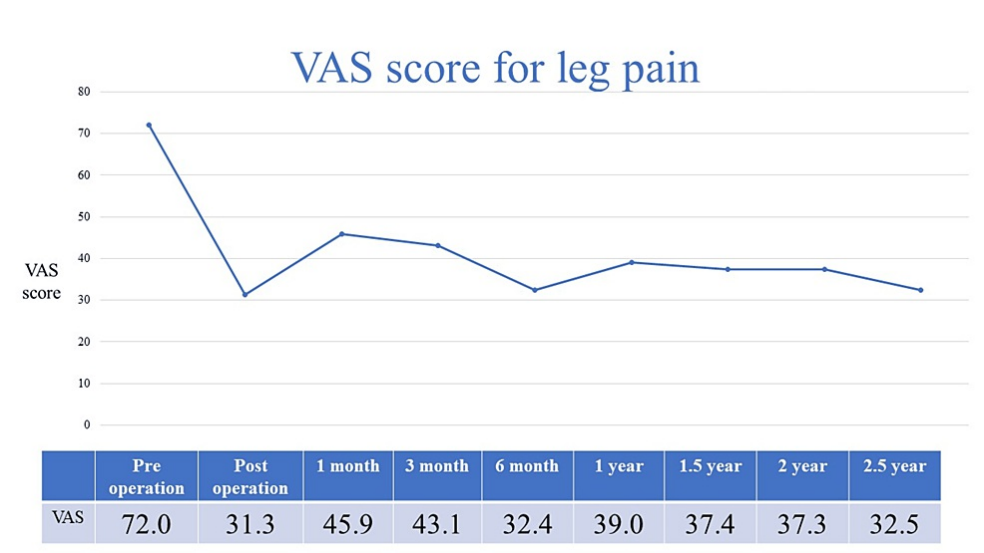
VAS score for leg pain

Immediately after TSCP, the average VAS score for back pain improved from 58.2 to 29.3, and for leg pain from 72.0 to 31.3 (Figures 1, 2).

**Figure 3:**
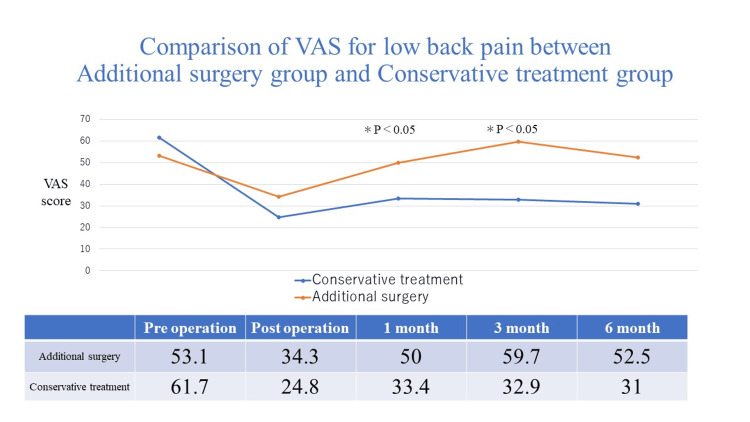
Comparison of VAS for low back pain between additional surgery group and conservative treatment group

Two years after TSCP, the average VAS score for back pain increased slightly and the average score for leg pain remained almost the same. Additional surgery was performed in 37 of 120 (31%) patients who underwent TSCP. The additional surgery group had significantly worse back pain at one and three months postoperatively than the conservative treatment group (Figure 3).

**Figure 4:**
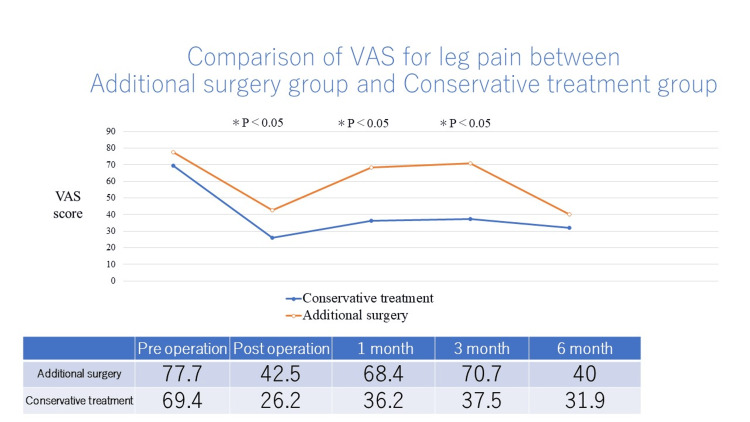
Comparison of VAS for leg pain between additional surgery and conservative treatment group

The additional surgery group had significantly worse leg pain immediately after TSCP and at 1 and 3 months postoperatively than the conservative treatment group (Figure 4).

Additional surgeries were performed at one month, three months, six months, one year, and 1.5 years postoperatively in five, 16, 11, three, and two cases, respectively. The follow-up rate was 96.6% (116/120 cases) at one year postoperatively. Logistic regression analysis demonstrated that a decreased spinal canal area (OR 0.986, p = 0.039) was associated with additional surgery (Table 1).

**Table 1:** Logistic regression analysis demonstrated that a decreased dural sac cross-sectional area (OR 0.986, p = 0.039) was associated with additional surgery

	Odds	P-value	95% confidence intervals
Age	0.997	0.902	0.951-1.046
Gender	0.812	0.643	0.336-1.960
Nerve root block	0.687	0.472	0.246-1.914
History of lumber surgery	0.355	0.094	0.106-1.191
Intermittent claudication	0.912	0.872	0.297-2.799
Spondylolisthesis	1.401	0.562	0.447-4.387
Scoliosis	1.212	0.677	0.490-2.999
Dural sac cross-sectional area	0.986	0.039	0.973-0.999

Age, gender, selective nerve root block, history of lumbar surgery, intermittent claudication, spondylolisthesis and scoliosis were did not associated with additional surgery. And as for complications, there were two cases of high spinal anesthesia. There were no infections and dural injuries.

## Discussion

The myeloscope and epidural approach has been developed since the 1930s. It was Baumann who first performed an arthroscopic evaluation of the spinal cord in a cadaveric donation [12]. He was able to see the spinal cord, the dura mater, and some of the nerve constituents of the cauda equina [12]. Racz et al. presented a new epidural catheter for injection of phenol [13]. New epidural catheters made of spiral stainless-steel coils coated with fluoropolymers were developed. Five patients with intractable pain were treated using this catheter [13]. Saberski reported an epiduroscopy technique with a lysis procedure for L5 radiculopathy [14]. The 0.8 mm diameter fiberoptic scope was introduced into the catheter through a valve. The catheter and fiberoptic were advanced cephalad, caudad, and rotated clockwise and counterclockwise. The L5 nerve root was visualized [3].

In this study, both back and leg pain improved after TSCP, and the clinical course was still good for two years. Immediately after TSCP, the average VAS score for back pain improved from 58.2 to 29.3, and for leg pain from 72.0 to 31.3. Two years after TSCP, the average VAS score for back pain increased slightly and the average score for leg pain remained almost the same. Veihelmann et al. conducted a prospective randomized blinded clinical trial to investigate whether epidural neuroplasty is superior to conservative treatment with physiotherapy [15]. Three months later, VAS scores for back and leg pain decreased in the epidural neuroplasty group. Analgesic medications also decreased in both groups. In addition, the VAS and Oswestry Disability Index (ODI) for back and leg pain were significantly better until 12 months postoperatively than in the conservatively treated group. Gerdesmeyer et al. analyzed the clinical efficacy of percutaneous epidural lysis for chronic nerve root pain in a randomized controlled trial [16]. ODI and VAS scores, as well as ODI versus VAS success rates, were better in the lysis group than in the control group at three, six, and 12 months. ODI in the lysis group improved from 55.3 ± 11.6 to 26.4 ± 10.8 at three months, while in the placebo group, it improved from 55.4 ± 11.5 to 41.8 ± 14.6 (P < 0.01). VAS improved from 6.7 ± 1.1 to 2.9 ± 1.9 in the active group and from 6.7 ± 1.1 to 4.8 ± 2.2 (P < 0.01) after placebo. Funao et al. conducted a multicenter study to evaluate the clinical outcomes of TSCP between patients with and without failed back surgery syndrome [8]. Eighty patients underwent lumber surgery (F group), and 191 have not had lumbar spinal surgery (N group). VAS scores for back pain in group N were 51 mm, 24 mm, 33 mm, 30 mm, and 30 mm preoperatively, immediately postoperatively, and at one, three, and six months postoperatively; VAS scores for leg pain in group N were 69 mm, 28 mm, 39 mm, 36 mm, and 32 mm. VAS scores for back pain in group F were 52 mm, 26 mm, 34 mm, 36 mm, and 36 mm preoperatively, immediately postoperatively, and at one, three, and six months postoperatively; VAS scores for leg pain in group F were 67 mm, 27 mm, 41 mm, 43 mm, and 40 mm. VAS scores for back and leg pain were clearly improved in both the N and F groups (p<0.01).

In this study, additional surgery was performed in 37 of 120 (31%) patients who underwent TSCP. In the additional surgery group, the average VAS score for back pain worsened one month after TSCP. The additional surgery group had poor improvement in average VAS score for leg pain immediately after TSCP compared to the conservative treatment group. If low back or leg pain recurs one month after TSCP, lumbar surgery may be required thereafter. After a 12-month follow-up of patients with chronic lumbar radicular pain undergoing percutaneous epidural lysis, additional lumbar surgery was performed in one of 46 patients [8]. Cho et al. reported clinical outcomes of consecutive patients with single-level lumbar disc herniation who underwent percutaneous epidural neuroplasty [17]. After epidural neuroplasty, subsequent surgery was required for 59 of 430 (13.7%) patients. After three-month follow-up of patients with lumbar foraminal spinal stenosis undergoing percutaneous transforaminal adhesiolysis, additional lumbar surgery was performed in one of 35 patients [18].

Logistic regression analysis demonstrated that a decreased cross-sectional spinal canal area (OR 0.986, p = 0.039) was associated with additional surgery. Age, gender, selective nerve root block, history of lumbar surgery, intermittent claudication, spondylolisthesis, and scoliosis were not associated with additional surgery. There is disagreement regarding the correlation between the cross-sectional spinal canal area and clinical outcomes after percutaneous epidural neuroplasty. Ji et al. reported that the efficacy of percutaneous epidural neuroplasty was not related to the dural sac area in one level of disc disease [19,20]. Park et al. reported that the dural sac area did not correlate with the efficacy of percutaneous adhesiolysis at one level.

A limitation of this study is the lack of controls. No imaging evaluation after TSCP was performed.

## Conclusions

We reviewed the outcomes of TSCP at our hospital. The average VAS score for back pain and leg pain improved. The number of elderly patients is increasing. Elderly patients may not be able to be treated with general anesthesia. Therefore, TSCP, which can be performed under local anesthesia, may be useful. However, 31% of patients who underwent TSCP required additional surgery. Patients who underwent TSCP and have a recurrence of symptoms one month after surgery may require additional surgery. It was found that the cross-sectional spinal canal area was a major factor in the need for additional surgery. It is important to understand which patients have difficulty improving their symptoms with TSCP.
